# Editorial: Tribute to Dr. Welda LaJean Chaffin - a legendary woman in fungal research

**DOI:** 10.3389/fcimb.2022.1115382

**Published:** 2022-12-19

**Authors:** Priya Uppuluri, Concha Gil, José Luis López-Ribot

**Affiliations:** ^1^ Division of Infectious Disease, The Lundquist Institute for Biomedical Innovation at Harbor-UCLA Medical Center, Torrance, CA, United States; ^2^ David Geffen School of Medicine, University of California (UCLA), Los Angeles, CA, United States; ^3^ Department of Microbiology and Parasitology, Faculty of Pharmacy, Complutense University of Madrid (UCM) and Ramon y Cajal Health Research Institute (IRYCIS), Madrid, Spain; ^4^ Department of Molecular Microbiology & Immunology, and South Texas Center for Emerging Infectious Diseases, The University of Texas at San Antonio, San Antonio, TX, United States

**Keywords:** LaJean Chaffin, woman in science, *Candida albicans*, legend, biofilm, cell wall

The goal of this Research Topic was to highlight the research heritage of Dr. LaJean Chaffin, a trailblazing woman scientist and an accomplished mentor, whose work laid the foundation in research on cell surface proteins and biofilm formation in fungal pathogens. This topic attracted 6 articles that have been viewed over 13,000 times as of December 2022.

## Adhesins and cell wall proteins in *Candida* spp.

Two articles from Oh et al. used bioinformatics and advanced 3D modeling to accomplish a better understanding of two families of *Candida* cell wall proteins – ALS and PIR. *C. albicans* Als family of proteins are adhesins that promote interactions between fungi and host cells, adhesion on medical devices and contribute to polymicrobial interactions. In their article, Oh et al. presented a comparative view of the ALS family across a broader expanse of the phylogenetic tree, and discovered that this important family of proteins is highly diverse and present in several fungi. Two novel takeaways from this study were, first, the identification of *S. cerevisiae* Sag1 as a new Als family protein. Second, the finding that *ALS* genes are located at the subtelomeric region in multiple species of budding yeasts, perhaps a reason for the high rates of genomic rearrangement in the *ALS* gene family. The authors though mention caveats in their study relating to allelic variability and differences in *ALS* gene expression at diverse growth stages and morphologies, which could contribute to variability in the assembly of the sequences. This they suggest could be resolved by testing allelic variability and expression of *ALS* genes spanning a larger number of fungal isolates and growth conditions.

Keeping with the same theme, Kim et al. explored the presence and function of another family of *Candida* cell wall proteins called Pir proteins (proteins with internal repeats). These proteins are linked to cell wall β-1,3-glucan and important for cell wall integrity in *Saccharomyces cerevisiae*. The authors constructed double mutants of the only two annotated homologues of the genes in *C. albicans* - *PIR1* and *PIR32*. When the phenotype was found to be dispensable, the authors probed further for the possibility at identifying additional genes that may be the *PIR* counterparts, also functionally. Indeed, bioinformatic analysis that spanned 16 fungal species, identified 75 proteins which harbored another consensus motif, considered a Pir-associated signature. The study further highlighted an elaborate expansion of Pir proteins especially in *C. albicans* and *C. dubliniensis* and their role in chlamydospore formation. Unlike *ALS* gene family, allelic variations in *PIR* was found to be quite limited. Indeed, these two articles highlighted the complex nature of cell wall associated gene families in fungi, opening up multiple hypothesis for testing their undisputed relevance in fungal biology and virulence.

Another interesting report that was included in this research collection was from Fernandez-Pereira et. al., who identified a completely novel adhesin gene in the non-filamenting pathogenic yeast *C. glabrata*. A high biofilm forming strain of *C. glabrata* isolated from central venous catheter infection was found to incorporate several proteins, not detected in the reference ATCC strain. Three of these proteins Epa22, Awp14, and Awp2e were novel. While knocking out *AWP14* yielded an unremarkable phenotype as it relates to biofilm growth, it did show diminished chitin binding and defective cell-cell interactions. Certainly, discovery of these novel proteins in clinical isolates require further functional characterization studies to elucidate their contribution in biofilm formation. Additionally, the study highlights the disparity in the dynamics of cell wall proteins between clinical isolates with exaggerated virulence phenotypes, and laboratory strains.

The fundamental contributions by Dr. LaJean Chaffin in the area of *C. albicans* cell wall biochemistry, was underlined in the review article by Chen et. al. The cell wall of *C. albicans* is a layered structure, consisting of an outer mannan layer that covers a central layer of the core structural polysaccharides β (1,3)-glucan and β(1,6)-glucan, as well as a basal chitin layer. The β(1,3)-glucan are an important drug target and an immunogenic epitope recognized by multiple host pattern recognition receptors. However, this epitope is masked by glycosylating proteins, and exposure of ß(1,3)-glucans hold tremendous promise in discovery of antifungal drugs and therapeutic antibodies alike. In their review, Chen et. al. enlisted an impressive array of environmental conditions and signaling pathways that regulate exposure in *C. albicans*, a highly immunogenic epitope. Interestingly, as a process of adaptation in the host, fungal pathogens avoid being recognized by immune cells by concealing (masking) β(1,3)-glucan. The environmental triggers for this include hypoxia, exposure to L-lactate, and iron limitation. On the other hand acidic pH of 4 or below enhances masking and triggers phagocytosis of *C. albicans* by neutrophils and macrophages, and pro-inflammatory cytokine production by PBMC’s. The review highlighted the role of Hog1 mediated neutrophil NET response as an important reason behind ß(1,3)-glucan exposure during infection, with chitin deposition playing the role of an accomplice in the process of unmasking. Other signaling pathways included the prominent role of Map kinase pathways (MAPK), namely Cek1 MAPK and Mkc1 MAPK in the phenomenon. Overall, harnessing the approach of unmasking ß(1,3)-glucan could pose as a therapeutic approach during mucosal infections, especially in oropharyngeal candidiasis, where the unmasked cells are specifically recognized oral epithelial receptors.

## Mixed species biofilms and novel pipeline antifungal drug

*C. albicans* frequently co-exists with various bacterial species at mucosal sites as a part of the normal flora. The article by Short et. al., elaborate that during infection, when the cell densities rise, this interaction becomes exaggerated and can manifest into beneficial symbiosis between species, such as increased drug resistance, virulence and biofilm formation. Two *C. albicans* proteins Als3 and Ece1 are described to be crucial for *C. albicans* virulence and mono-species biofilm growth. In the current study the authors describe the importance of these genes also in a mixed species biofilm composed of *C. albicans* and *Staphylococcus aureus*. Corresponding to previously published reports, Short et. al. also found that *S. aureus* binds to *C. albicans* hyphae *via* Als3, while Ece1 was unessential for mixed species biofilm growth. However, loss of Ece1 was compensated by an array of virulence genes triggered by *S. aureus*.

There is a dearth of antifungals to combat pathogenic fungi, due to increase in the evolution of resistance, and biofilm growth on medical devices. The comprehensive study by Quindos et. al., investigates the efficacy of a new drug Ibrexafungerp (SCY-078), a novel antifungal targeting the fungal cell wall, and comparator drugs against a collection of 434 European blood isolates of several different *Candida* species. Ibrexafungerp MIC’s were either similar to or lower than the comparator drugs and the drug was efficacious also against fluconazole and echinocandin resistant isolates. Overall, similar to that reported against bloodstream isolates recovered from American patients, the drug was highly efficacious against both albicans and non-albicans *Candida* isolates from European patient population.

## Conclusion

This collection of studies highlights the advancements in cell wall and biofilm research that is built on the solid foundation laid by exemplary scientists such as Dr. LaJean Chaffin. We believe, this Research Topic serves as an excellent tribute to senior investigators in the field, who made an indelible mark in science and in the small yet significant mycology research community.

## Author contributions

PU wrote the manuscript, CG and JR edited the manuscript. All authors contributed to the article and approved the submitted version.

